# The Chemical and Antibacterial Evaluation of St. John's Wort Oil Macerates Used in Kosovar Traditional Medicine

**DOI:** 10.3389/fmicb.2017.01639

**Published:** 2017-09-08

**Authors:** James T. Lyles, Austin Kim, Kate Nelson, Angelle L. Bullard-Roberts, Avni Hajdari, Behxhet Mustafa, Cassandra L. Quave

**Affiliations:** ^1^Center for the Study of Human Health, Emory University Atlanta, GA, United States; ^2^Department of Dermatology, Emory University School of Medicine Atlanta, GA, United States; ^3^Department of Biology, University of Pristina Prishtinë, Kosovo; ^4^Emory Antibiotic Resistance Center, Emory University Atlanta, GA, United States

**Keywords:** *Hypericum perforatum*, *Staphylococcus aureus*, traditional medicine, phototoxicity

## Abstract

*Hypericum perforatum* L. (Hypericaceae), or St. John's Wort, is a well-known medicinal herb often associated with the treatment of anxiety and depression. Additionally, an oil macerate (Oleum Hyperici) of its flowering aerial parts is widely used in traditional medicine across the Balkans as a topical wound and ulcer salve. Other studies have shown that Oleum Hyperici reduces both wound size and healing time. Of its active constituents, the naphthodianthrone hypericin and phloroglucinol hyperforin are effective antibacterial compounds against various Gram-positive bacteria. However, hyperforin is unstable with light and heat, and thus should not be present in the light-aged oil macerate. Additionally, hypericin can cause phototoxic skin reactions if ingested or absorbed into the skin. Therefore, the established chemistry presents a paradox for this *H. perforatum* oil macerate: the hyperforin responsible for the antibacterial bioactivity should degrade in the sunlight as the traditional oil is prepared; alternately, if hypericin is present in established bioactive levels, then the oil macerate should cause photosensitivity, yet none is reported. In this research, various extracts of *H. perforatum* were compared to traditional oil macerates with regards to chemical composition and antibacterial activity (inhibition of growth, biofilm formation, and quorum sensing) vs. several strains of *Staphylococcus aureus* in order to better understand this traditional medicine. It was found that four Kosovar-crafted oil macerates were effective at inhibiting biofilm formation (MBIC_50_ active range of 0.004–0.016% v/v), exhibited moderate inhibition of quorum sensing (QSIC_50_ active range of 0.064–0.512% v/v), and contained detectable amounts of hyperforin, but not hypericin. Overall, levels of hypericin were much higher in the organic extracts, and these also exhibited more potent growth inhibitory activity. In conclusion, these data confirm that oil macerates employed in traditional treatments of skin infection lack the compound credited with phototoxic reactions in *H. perforatum* use and exhibit anti-biofilm and modest quorum quenching effects, rather than growth inhibitory properties against *S. aureus*.

## Introduction

*Hypericum perforatum* L. (Hypericaceae), or St. John's Wort, is a well-known medicinal herb regularly associated with the treatment of anxiety, historically determined by the ancient Greek physicians Pliny and Hippocrates (Blumenthal, 2002). The plant grows up to 1 m in height and features yellow flowers, rounded leaves, and oblong petals populated with a number of brown-black glandular dots, giving the plant its eponymous “perforated” appearance (WHO, 1999), Figure 1. It is native to Europe and Asia, while also having spread as an invasive species in North America and Oceania. Often used to treat depression and other mood disorders (Ng et al., 2017), dietary supplements featuring St. John's Wort reached an annual sale of $6 million in the United States in 2015 (Smith et al., 2016). *H. perforatum* is well-characterized chemically: many secondary metabolites have been identified, including naphthodianthrones (hypericin), phloroglucinols (hyperforin), flavonoid glycosides (hyperoside), biflavones, and anthocyanidins (Porzel et al., 2014). Currently, many compounds are now understood in mechanism and function; for example, antidepressant activity has been attributed to hyperforin, which acts as a reuptake inhibitor for neurotransmitters such as dopamine, norepinepherine, serotonin, and glutamate (Chatterjee et al., 1998) and is now used in the standardization of many *Hypericum*-based products sold on the commercial market (USP, 2015).

**Figure 1:**
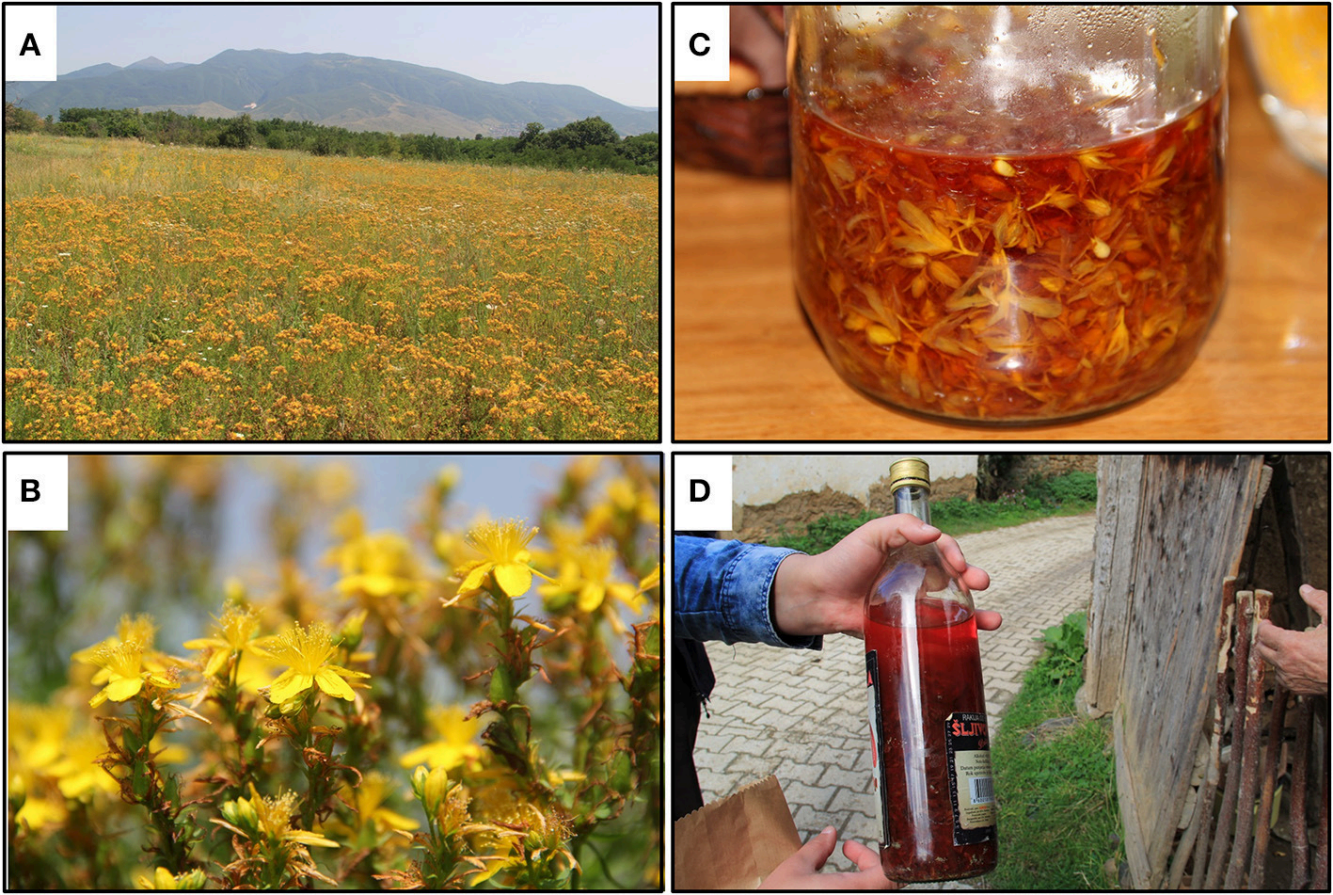
Traditional production of *H. perforatum* oil macerates for topical applications. **(A)** Plants are collected from the wild. **(B)** The flowering aerial parts are harvested. **(C)** The flowers are covered with oil (typically olive oil or sunflower oil) and exposed to the sun for 40 days. **(D)** The oil is ready for use once it has passed the sun exposure period and has taken on a blood red color.

Additionally, an oil macerate of *H. perforatum* flowers (Oleum Hyperici) is widely used as a traditional remedy across Bosnia and Herzegovina, Turkey, and Serbia for skin ulcers and burns (Kültür, 2007; Redžić, 2007; Šarić-Kundalić et al., 2010; Savikin et al., 2013), as well as in Kosovo (Mustafa et al., 2012a,b, 2015). The documentation of continued usages of the oil across the Balkan Peninsula stands in contrast to its less common use in the West. Historic literature indicates a past appreciation for the oil; *The Eclectic Dispensatory of the United States of America* quoted it as being “a fine red balsamic ointment for wounds, ulcers, swellings, tumors, etc.” in 1852 (King, 1852), and *Potter's Cyclopaedia of Botanical Drugs and Preparations* described it as “a healing application to wounds, sores, ulcers, and swellings” to Londoners in 1907 (Wren, 1907). Recent research showed that polysaccharides from *H. perforatum* have antimicrobial activity against *Escherichia coli, Shigella dysenteriae, Salmonella typhi, Bacillus cereus*, and *Staphylococcus aureus* when tested by a disk diffusion assay (Heydarian et al., 2017). Another study demonstrated *H. perforatum* extracts and partitions had antibacterial activity against several oral bacteria including *Streptococcus mutans, S. sobrinus, Lactobacillus plantarum*, and *Enterococcus faecalis* (Suntar et al., 2016).

**Figure 2:**
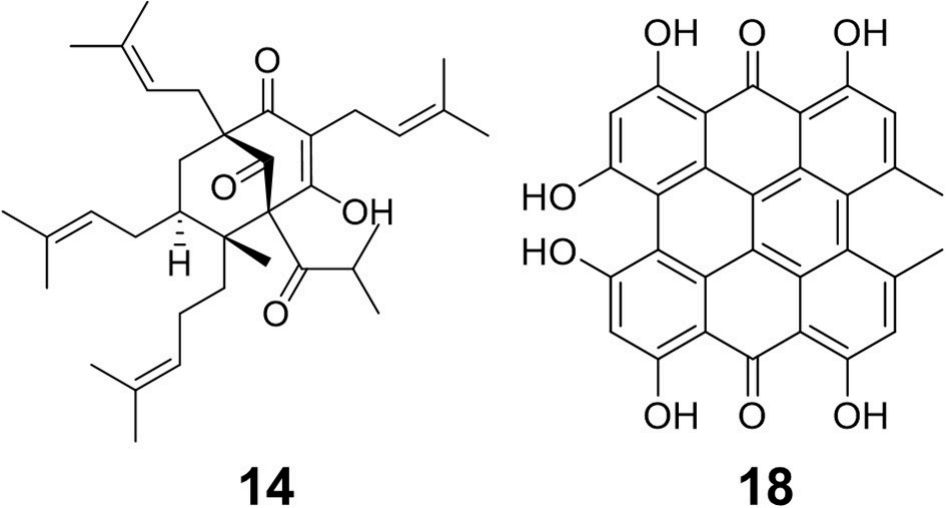
Structures of two bioactive compounds, hyperforin **(14)** and hypericin **(18)**, examined in our chemical analysis of the *H. perforatum* extracts and traditional formulas.

Oleum Hyperici is prepared in the Balkan region by covering the flowering aerial parts of the plant in olive or sunflower oil in a transparent container (glass or plastic) and exposing to sunlight for at least 40 days until it turns an intense red color (Mattalia et al., 2013), Figure 1. Recent research has shown that Oleum Hyperici reduces both external wound size and healing time as an antibacterial (Suntar et al., 2010). The phloroglucinol hyperforin and naphthodianthrone hypericin (Figure 2) are reported to be responsible for its antibacterial activity (Saddiqe et al., 2010), effective as antibacterial compounds against various Gram-positive bacteria (Schempp et al., 1999), including *Bacillus subtilis, E. faecalis, Staphylococcus epidermidis*, and *Micrococcus luteus* (Marcetic et al., 2016), while ineffective against Gram-negative bacteria (Gibbons et al., 2002). Many compounds other than the naphthodianthrone and phloroglucinol derivatives have been identified in *H. perforatum* flowers; including the flavonol hyperoside, biflavonols such as amentoflavone and biapigenin, low levels of xanthone derivatives, common phenolic acids such as caddeic acid, chlorogenic acid, and ferulic acid, tannins and catechin derivatives are also present at significant concentrations (Dostalek and Stark, 2012; Matei et al., 2015).

Nevertheless, phloroglucinols are quite unstable with light and heat (Orth et al., 1999; Ang et al., 2002), and thus should not be present in an aged oil macerate of *H. perforatum*. Hyperforin, specifically, is susceptible to oxidation into furohyperforin, which is completely ineffective when treating Gram-positive and Gram-negative bacteria (Miraldi et al., 2006). Additionally, hypericin can cause phototoxic skin reactions if ingested or absorbed into the skin (Kamuhabwa et al., 1998), known as “hypericism”—a common pharmaceutical warning for fair-skinned individuals taking *Hypericum* supplements or evidenced by livestock that develop extreme photosensitivity after grazing on *H. perforatum* flowers (Genter, 2001)—as UV-activated hypericin has been demonstrated to kill human keratinocytes and melanocytes by means of necrosis and apoptosis (Davids et al., 2008). While hypericin is often cited as the main photosensitizing agent, pseudohypericin and hyperforin may also contribute to the phototoxicity of *H. perforatum* preparations (Onoue et al., 2011). Although hypericin in a *H. perforatum* extract or in combination with other compounds found in *H. perforatum*, such as chlorgenic acid, can exhibit a lower phototoxicity than the hypericin alone (Schmitt et al., 2006).

Therefore, the established chemistry presents an interesting paradox to this traditional preparation of *H. perforatum*. The hyperforin thought to be responsible for much of the antibacterial bioactivity should degrade in the sunlight as the remedy is prepared, unless the oil increases the stability of certain terpenoids as it is known to do (Boskou, 1978) that, in turn, act as sacrificial antioxidants for hyperforin. Alternately, if hypericin is present in established bioactive levels and contributes to the oil's red colorization, then the traditionally prepared oil macerate should cause photosensitivity, yet none is reported. Here, we approach two central questions: (1) Do traditionally formulated Oleum Hyperici from the Balkans contain the phototoxic compound, hypericin; and (2) Does the anti-staphylococcal activity of various *H. perforatum* formulations differ? Based on a lack of skin sensitization reports following use of the oil (data from previous field studies in the Balkans) and its traditional use in treating skin infection, we hypothesize that Oleum Hyperici does not contain hypericin, but does exhibit antibacterial activity against the most common cause of skin infection, *S. aureus*. In this research, organic and aqueous extracts of *H. perforatum* as well as commercial dietary supplements (tablet and liquid tincture) were compared to four Kosovar oil macerates in both chemical composition and antibacterial activity against *S. aureus* in order to investigate the contradictory nature of this traditional medicine.

## Experimental methods

### Collection and extraction of *H. perforatum* samples

Plant material was collected following standard guidelines for collection of wild specimens (WHO, 2003), while *H. perforatum* olive oil, sunflower oil, unknown oil A, and unknown oil B macerates were procured from local Prizren, Kosovo markets and households in this region by CQ and AH. All procured oils were reported to have been created in the traditional method, with the flowering aerial parts covered in vegetable oil and exposed to sunlight for 40 days. Voucher specimens were deposited at the Emory University Herbarium (Accession Numbers: 20051 and 20091) and University of Prishtina Herbarium (BP-0002); digital copies of the specimens are accessible for viewing online via the SERNEC web portal (SERNEC, 2017). St. Johns Wort dietary supplements were purchased from a commercial vendor in Atlanta, Georgia, USA.

Shade-dried, aerial parts of *H. perforatum* were ground in a Thomas Scientific Wiley Mill (Swedesboro, NJ) through a 2 mm mesh. Dry powder (75 g) was transferred into an amber bottle and macerated in 750 mL of methanol (MeOH). The jug was wrapped in aluminum foil and left in the dark for 48 h, with agitation every 24 h. The MeOH extract was filtered through coarse and then fine filter paper. The marc was returned to the bottle and underwent a second 48 h maceration with 750 mL of MeOH and filtered as before. The filtrates were combined and evaporated using a rotary evaporator at <40°C. The dried extract was re-dissolved in DI water, shell frozen, and freeze-dried overnight on a Labconco FreeZone 2.5 lyophilizer (Kansas City, MO). All solvents were acquired from Fisher Chemical, Certified ACS grade (Pittsburg, PA). The dry extract was scraped and transferred to scintillation vials wrapped in aluminum foil, and then stored at −20°C. All of the above procedures were conducted under aluminum foil tents to reduce ambient light exposure. An aqueous extract of the same material was also obtained by boiling 40 g of ground plant material in 400 mL DI water. This decoction was then strained through cheese cloth, double filtered, concentrated, freeze dried, and collected similarly to the above organic extract.

The commercial St. John's Wort dietary supplement extract was indicated to contain 300 mg of St. John's Wort Extract (flower) per capsule standardized to 0.3% hypericin. Three of these capsules were emptied and pooled, with 1.1 g of the contained powder from three capsules dissolved in 4.5 mL of MeOH in an Eppendorf tube. The solution was sonicated under aluminum foil for 30 min and centrifuged at 3,000 rpm for 15 min. The supernatant was transferred to a round-bottom flask and concentrated by removal of MeOH with a rotary evaporator. A 10 mL aliquot of the commercial liquid St. John's Wort flower buds and tops tincture (65–75% USP alcohol, indicating a 500 mg mL^−1^ herb equivalency) was concentrated with a rotary evaporator. Both commercial extracts were concentrated, stored and tested under reduced light. In total, eight samples were prepared for this experiment: a MeOH extract of the aerial parts (MAP), a MeOH extract of a tablet supplement (TS), a concentration of an EtOH liquid supplement (LS), a decoction of aerial parts (HAP), an olive oil macerate (OOO), a sunflower oil macerate (SOO), a macerate of unknown oil A (UOA), and another macerate of unknown oil B (UOB), Table 1. Extracts were prepared for bacterial assays by dissolving in DMSO at 10 mg mL^−1^. Oil macerates and an olive oil control were prepared by first mixing with Tween20 (4:1), and then further diluting in media relevant to each bioassay (1:4), for a final 16% v/v oil emulsion. Tea tree essential oil was prepared at 10% v/v oil in an emulsion mixture (0.5% v/v Tween80 in DMSO) for use as a positive control.

**Table 1:** Description of extracts made from the flowering aerial parts of *Hypericum perforatum*.

**Extract abbreviation**	**Extraction material**	**Extraction solvent**	**Extraction method**	**Percent yield**	**Vehicle**
MAP	Flowering Aerial Parts	Methanol	Maceration	28%	DMSO
TS	Tablet Supplement (“from flower”)	Methanol	Sonication	74%	DMSO
LS	Liquid Supplement (from “flower buds and tops”)	Already in EtOH	Rotary evaporation only	19%	DMSO
HAP	Flowering Aerial Parts	DI Water	Decoction	14%	DMSO
OOO	Flowering Aerial Parts	Olive Oil	40 Days in Sun	–	Tween20 + relevant media
SOO	Flowering Aerial Parts	Sunflower Oil	40 Days in Sun	–	Tween20 + relevant media
UOA	Flowering Aerial Parts	Unknown Oil A	40 Days in Sun	–	Tween20 + relevant media
UOB	Flowering Aerial Parts	Unknown Oil B	40 Days in Sun	–	Tween20 + relevant media

*“–” denotes no % yield calculated on oil macerates acquired in Kosovo*.

### Characterization by HPLC and LC-FTMS

High performance liquid chromatography (HPLC) analysis was conducted on an Agilent Technologies ZORBAX Eclipse XDB-C18 250 × 4.6 mm, 5 μm column (Santa Clara, CA) with a compatible guard column at a column temperature of 25°C, monitored at 588 nm for extracts and 254 nm for oil macerates, adapting a method for a smaller column size by Liu (Liu et al., 2005). Runs were performed on an Agilent 1260 Infinity system using OpenLab CDS ChemStation. Reagents were HPLC grade and purchased from Fisher Scientific, with the exception of the Type 1 DI water, which was obtained from an EMD Millipore MILLI-Q water system (Billerica, MA). Mobile phases consisted of: (A) 20 mM ammonium acetate in water and ACN (9:1) and (B) ACN. The flow rate was 1 mL/min using samples at a 100 mg mL^−1^ concentration. Samples were prepared using HPLC and MS-grade MeOH, while oil macerates were analyzed after being dissolved in ethyl acetate (4:1). The injection volume was 5 μL, with gradient elution beginning at 50% B, increasing linearly from 2 to 22 min to 100% B, holding at this concentration for 9 min, before returning to initial conditions for 9 min.

The above parameters were also used for liquid chromatography Fourier transform mass spectrometry (LC-FTMS) experiments. Samples were run on a Shimadzu SIL-ACHT (Tokyo, Japan) and Dionex 3600SD HPLC pump (Sunnyvale, CA), with data acquired in MS mode scanning from an *m/z* of 150–1,500 on a Thermo Scientific LTQ-FT Ultra MS in both negative and positive ESI modes and processed with Thermo Scientific XCalibur 2.2 SP 1.48 software (San Jose, CA). The capillary temperature was 275.0°C, sheath gas of 60, source voltage of 5.0 kV and current 100.00 μA, and the capillary voltage −19.0 or +32.0 V, respectively. Peaks featuring putative compounds of the oil macerates were identified throughout the entire chromatogram and searched across the Dictionary of Natural Products (CRC Press) and Scifinder (Chemical Abstracts Service). High resolution masses of compounds were determined from the LC-FTMS data and searches conducted throughout the databases for all compounds identified in *H. perforatum* within a similar mass range. Additionally, an authentic standard of hyperforin DCHA (AdipoGen Corp., Sandiego CA) with ≥97% purity was analyzed by the previously described LC-FTMS method to aid in identification of this compound in the various *H. perforatum* preparations.

### UV-Vis analysis

An Agilent Cary 50 UV-Vis Spectrometer was used to obtain UV-Vis spectra from 190 to 1,100 nm of the Oleum Hyperici samples and an olive oil control sample (Badia Extra Virgin Olive Oil). The data was collected and spectra analyzed using WinUV and Microsoft Excel.

### Antibacterial evaluation

#### Growth inhibition assay

*S. aureus* cultures (described in Table 2) were grown in Tryptic Soy Broth (TSB), with cation-adjusted Mueller Hinton broth (CAMHB) used for minimum inhibitory concentration (MIC) evaluation following standard Clinical and Laboratory Standards Institute (CLSI) methods (CLSI, 2013). To present a detailed view of the growth inhibitory activity across all samples tested, the MIC_50_ and MIC_90_, defined as the concentration required for at least 50 or 90% inhibition of growth, were both reported. The MIC_90_ is equivalent to the “MIC,” defined as the concentration required for no visible growth in the well. All extracts and Oleum Hyperici samples prepared in this study were examined for MIC values against *S. aureus* strains representing the four accessory gene regulator (*agr*) alleles (*agr*I: AH1677, *agr*II: AH430, *agr*III: AH747, *agr*IV: AH1872) to observe potential trends in the inhibition of quorum sensing activity, MRSA strain LAC (AH1263), as well as a biofilm test strain (UAMS-1). All concentrations were tested in triplicate and repeated twice on different days. Controls included the vehicles (DMSO and olive oil emulsion), tea tree oil as an antibacterial oil control (Thursday Plantation, Australia), and antibiotics Vancomycin and Ampicillin (MP Biomedicals, Santa Ana, CA). Overnight cultures were standardized by optical density (OD) to 5 × 10^5^ CFU mL^−1^, and this was confirmed by plate counts of colonies. MIC_50_ and MIC_90_ values were assigned as described (Quave et al., 2015); this was determined by reading plates at an OD_600 nm_ in a Cytation 3 multimode plate reader (BioTek, Winooski, VT) after 18 h incubation. In addition, MIC values of oil macerates were determined on an oil macerate volume/final well volume% basis (compared to μg mL^−1^ reporting for organic and aqueous extracts).

**Table 2:** Summary of *S. aureus* strain characteristics.

**Designation**	**Characteristics**	**References**
UAMS-1	Methicillin-sensitive *S. aureus* (MSSA), osteomyelitis isolate, PFT USA200	Cassat et al., 2005
UAMS-929	*sarA* mutant of UAMS-1	Blevins et al., 2002; Beenken et al., 2003
AH1263	Community-associated Methicillin resistant *S. aureus* (CA-MRSA), PFT USA300, *agr* group I; erythromycin sensitive LAC	Boles et al., 2010
AH1677	*S. aureus agr* P3-YFP reporter, *agr* group I	Kirchdoerfer et al., 2011
AH430	*S. aureus agr* P3-YFP reporter, *agr* group II	Kirchdoerfer et al., 2011
AH1747	*S. aureus agr* P3-YFP reporter, *agr* group III	Kirchdoerfer et al., 2011
AH1872	*S. aureus agr* P3-YFP reporter, *agr* group IV	Kirchdoerfer et al., 2011

#### Quorum sensing inhibition assay

Quorum sensing activity was investigated as described (Quave and Horswill, 2014; Quave et al., 2015), using previously described (Kirchdoerfer et al., 2011) *S. aureus agr* P3-YFP reporter strains (AH1677, AH430, AH1747, AH1872; Table 2). Briefly, overnight cultures grown in TSB supplemented with chloramphenicol (Cam) were diluted in fresh media with Cam to yield a final well starting inoculum of 5 × 10^5^ CFU mL^−1^. Black sided microtiter plates (Costar 3603) were incubated at 37°C with shaking (1,200 rpm) in a Stuart SI505 incubator (Bibby Scientific, Burlington, NJ) with a humidified chamber. Readings at OD_600 nm_ and fluorescence (top reading, 493 excitation, 535 nm emission, gain 60) were taken after 18 h incubation. Controls included vehicles (DMSO and olive oil emulsion) and 224C-F2, a previously reported quorum sensing inhibitor (Quave et al., 2015). Inhibition of quorum sensing activity for oil macerates was determined on a volume/volume% basis (compared to μg mL^−1^ reporting for organic extracts). All extracts were tested at sub-inhibitory concentrations for growth, as determined in MIC assays.

To determine if any observed quorum sensing inhibition was influenced by potential growth inhibitory effects of the test agents, growth and fluorescence was monitored in parallel at multiple time points over a 20 h period. Furthermore, colony counts were taken at 18 h post incubation by serial diluting and plating aliquots of treatment and control groups in triplicate onto TSA using the drop-plate method (10 μL drops). Plates were incubated for 12 h, after which dilution factors with 3–30 colonies present per drop were counted to determine final CFU mL^−1^ for each group.

#### Biofilm inhibition assay

Anti-biofilm activity was investigated using a human plasma protein-coated assay as previously described (Beenken et al., 2010; Quave et al., 2012) with *S. aureus* strains UAMS-1 and its isogenic *sarA* mutant (UAMS-929) as a biofilm deficient phenotypic control (Beenken et al., 2003). Briefly, following inoculation and addition of media (containing extract or vehicle alone) with a starting inoculum of 5 × 10^5^ CFU mL^−1^, 96-well plates (Falcon 35-1772) were incubated for 22 h at 37°C, washed with phosphate-buffered saline (PBS), fixed with ethanol, stained with crystal violet and rinsed in tap water. The stain was then eluted into the ethanol and was transferred to a new plate prior to quantification of the eluate at an OD_595 nm_. The MBIC_50_ and MBIC_90_ are defined here as the minimum concentration of test agent required to inhibit 50 or 90% of biofilm formation, respectively. Controls included vehicles (DMSO and olive oil emulsion), tea tree oil as an oil comparison, and 220D-F2, a previously reported biofilm inhibitor (Quave et al., 2012).

### Statistical analysis

All tests were performed in triplicate and repeated on at least two different occasions. Heteroscedastic Student's *t-*tests were performed in Microsoft Excel and significance is denoted for all tests at ^*^P < 0.05, ^†^*P* < 0.01, and ^‡^*P* < 0.001.

## Results

### Growth inhibition

**Figure 3:**
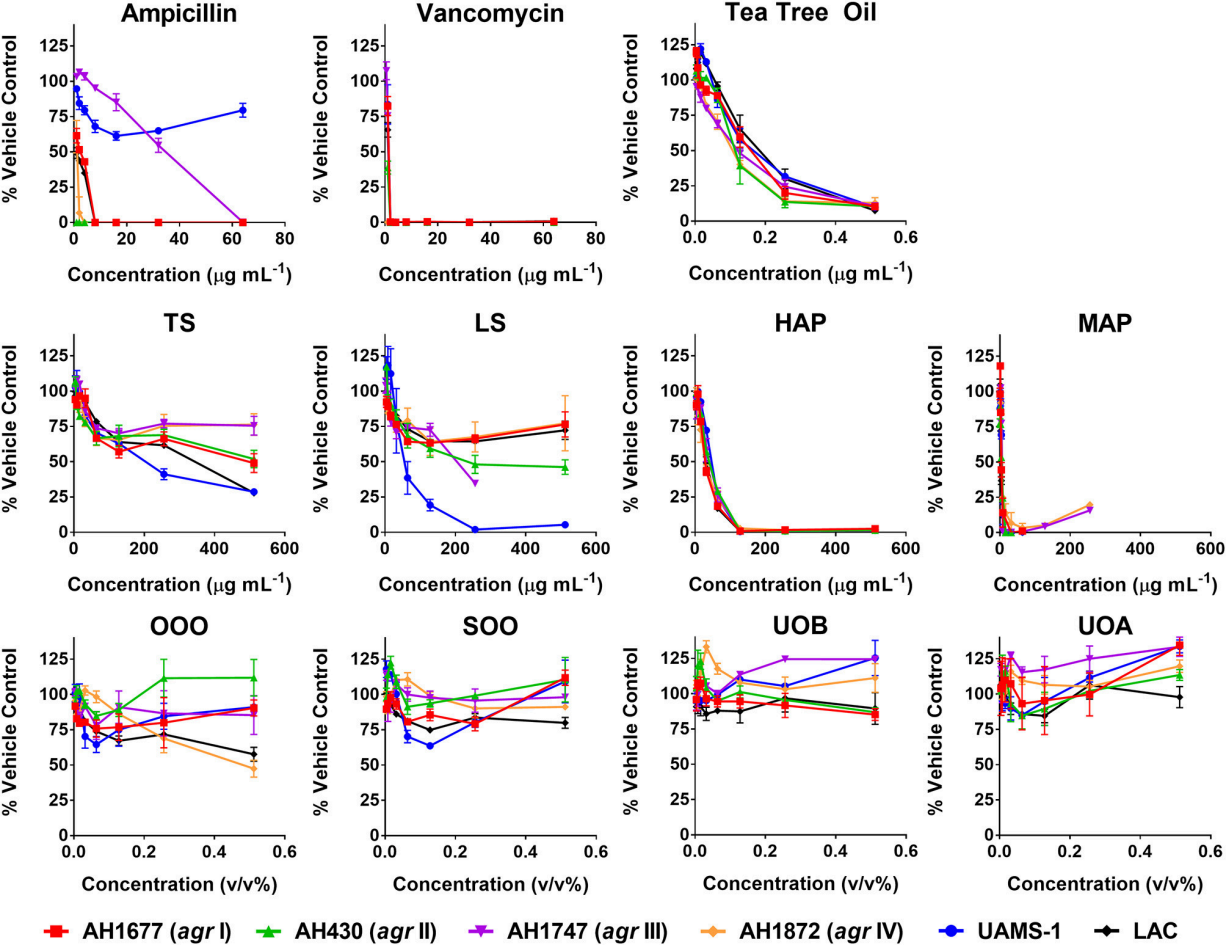
Growth inhibitory activity of *H. perforatum* extracts against strains of *S. aureus*. Activity was detected by measure of optical density of wells (OD_600nm_) and is reported as percent of the vehicle control. Positive controls included antibiotics (ampicillin and vancomycin) and tea tree oil.

**Figure 4:**
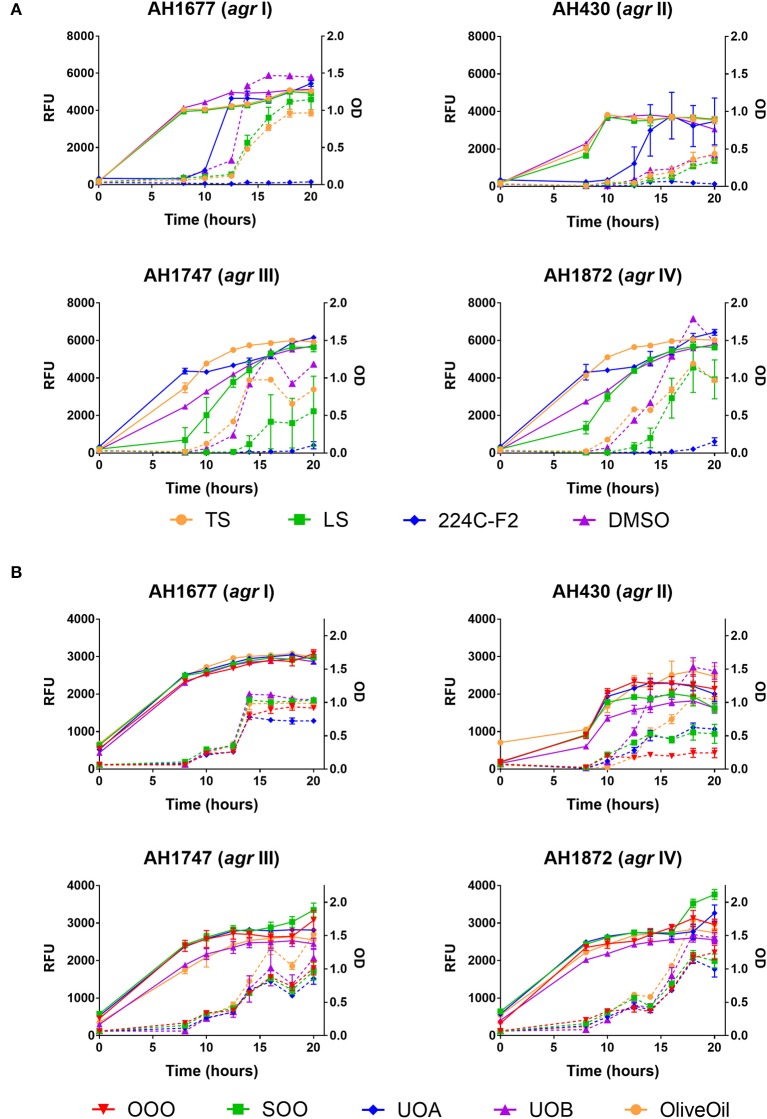
Impact of *H. perforatum* commercial supplements and Oleum Hyperici samples on growth and *agr* activity over 20 h as determined by optical density and fluorescence measures of four *S. aureus agr* reporters. Solid lines in the graphs denote OD; broken lines denote RFU. **(A)** Organic extracts and controls. **(B)** Oil macerates and controls. Test concentrations correspond with results on quorum sensing inhibition reported in Table 3. Briefly, TS and LS were tested at 32 μg mL^−1^, DMSO (vehicle control) at eight concentrations from 0.02 to 0.64% v/v, and 224C-F2 (positive control) at eight concentrations from 0.5 to 64 μg mL^−1^, with results from the highest concentrations for the vehicle and positive control reported here. For oil macerate tests, OOO was tested at 0.512% v/v for *agr* I and III, 0.064% for *agr* II, and 0.256% for *agr* IV; SOO was tested at 0.512% for *agr* I, III, and IV, 0.064% for *agr* II; UOA was tested at 0.512% for *agr* I, III, and IV, 0.0128% for *agr* II; UOB was tested at 0.512% for *agr* I, III, and IV, 0.0128% for *agr* II. Lastly, Olive Oil was tested at 8 concentrations from 0.004 to 0.512% and the highest concentration is reported here.

**Figure 5:**
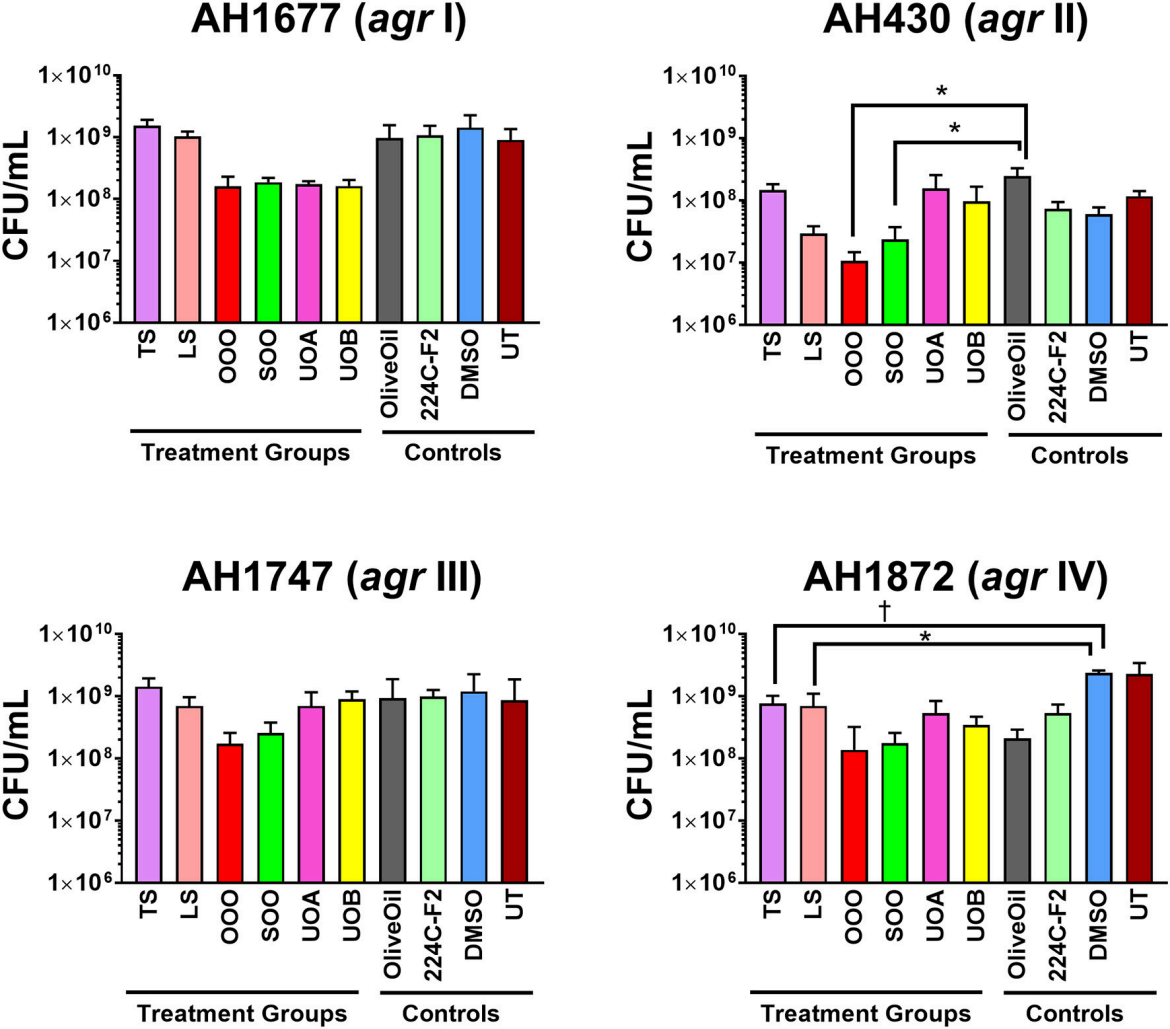
Impact of *H. perforatum* commercial supplements and Oleum Hyperici samples on *S. aureus* growth, as determined by colony counts. To further investigate whether the decreased level of quorum sensing activity in extracts and oil macerates was due to any potential growth inhibitory effects, the number of colony forming units (CFU) per mL of broth was determined at 18 h. Test concentrations correspond to those reported in Figure 4. Significant differences between the vehicle control and treatment groups are denoted as ^*^P < 0.05, ^†^*P* < 0.01, and ^‡^*P* < 0.001.

HAP (aqueous extract of flowering aerial parts; see Table 1) and MAP (methanol extract of flowering aerial parts) exhibited the strongest growth inhibitory activity across all *S. aureus* strains examined, with MIC_90_ values of 128 and 8–32 μg mL^−1^, respectively (Figure 3). The Oleum Hyperici samples (OOO, SOO, UOA, UOB; see Table 1) did not exhibit growth inhibitory action at the concentration range examined (maximum of 0.512% v/v), with the exception of OOO, which exhibited an MIC_50_ of 0.512% v/v for one strain (AH1872). The commercial supplements (LS—liquid supplement and TS—tablet supplement) likewise exhibited only limited growth inhibitory activity; LS exhibited a MIC_50_ in the range of 64–256 μg mL^−1^ against three of the strains, and a MIC_90_ of 256 μg mL^−1^ against one. Dose-response data is presented in Figure 3 and summary of MICs in Table 3. These findings concerning the lack of strong antibacterial activity by the commercial supplements (TS and LS) and Oleum Hyperici samples were also confirmed by multiple OD readings over a 20 h period (Figure 4) and colony counts (Figure 5).

**Table 3:** Summary of bioactivity data.

**Strain**	**Test**	**All reported in** μ**g mL**^**−1**^						**All reported in % v/v**					
		**MAP**	**TS**	**LS**	**HAP**	**Van**	**Amp**	**OOO**		**SOO**	**UOA**	**UOB**	**TTO**
UAMS-1	MIC_50_	8	256	64	64	1	–	–		–	–	–	0.256
	MIC_90_	16	–	256	128	2	–	–		–	–	–	0.512
	MBIC_50_	8	16	16	512	NT	NT	0.008		0.016	0.016	0.004	0.008
	MBIC_90_	16	64	128	–	NT	NT	–		–	–	–	–
AH1263	MIC_50_	4	512	–	32	1	0.5	–		–	–	–	0.256
	MIC_90_	16	–	–	128	2	8	–		–	–	–	0.512
AH1677 (*agr*I)	MIC_50_	4	512	–	32	1	4	–		–	–	–	0.256
	MIC_90_	16	–	–	128	2	8	–		–	–	–	0.512
	QSIC_50_	–	–	–	–	NT	NT	–		0.512	–	–	NT
AH430 (*agr*II)	MIC_50_	8	–	256	64	2	0.0625	–		–	–	–	0.128
	MIC_90_	16	–	–	128	2	0.125	–		–	–	–	0.512
	QSIC_50_	–	32	32	–	NT	NT	0.064		0.064	0.128	–	NT
AH1747 (*agr*III)	MIC_50_	8	–	256	64	1	32	–		–	–	–	0.128
	MIC_90_	8	–	–	128	2	32	–		–	–	–	0.512
	QSIC_50_	–	–	–	–	NT	NT	–		–	–	–	NT
AH1872 (*agr*IV)	MIC_50_	8	–	–	32	2	2	0.512		–	–	–	0.128
	MIC_90_	32	–	–	128	2	2	–		–	–	–	0.512
	QSIC_50_	–	–	–	–	NT	NT	0.256		0.512	0.512	0.512	NT
													

*Minimum inhibitory concentration (MIC) values determined for each sample vs. each strain in this study; Minimum biofilm inhibitory concentration (MBIC) values determined for UAMS-1; Quorum sensing inhibitory concentration (50%) values (QSIC_50_) determined using agr reporter strains. TTO, tea tree oil; Van, vancomycin; Amp, ampicillin; –, not detected; NT, not tested. All organic extracts and antibiotics reported in units of μg mL^−1^; all oil macerates and oil controls reported as % v/v*.

### Quorum sensing inhibition

**Figure 6:**
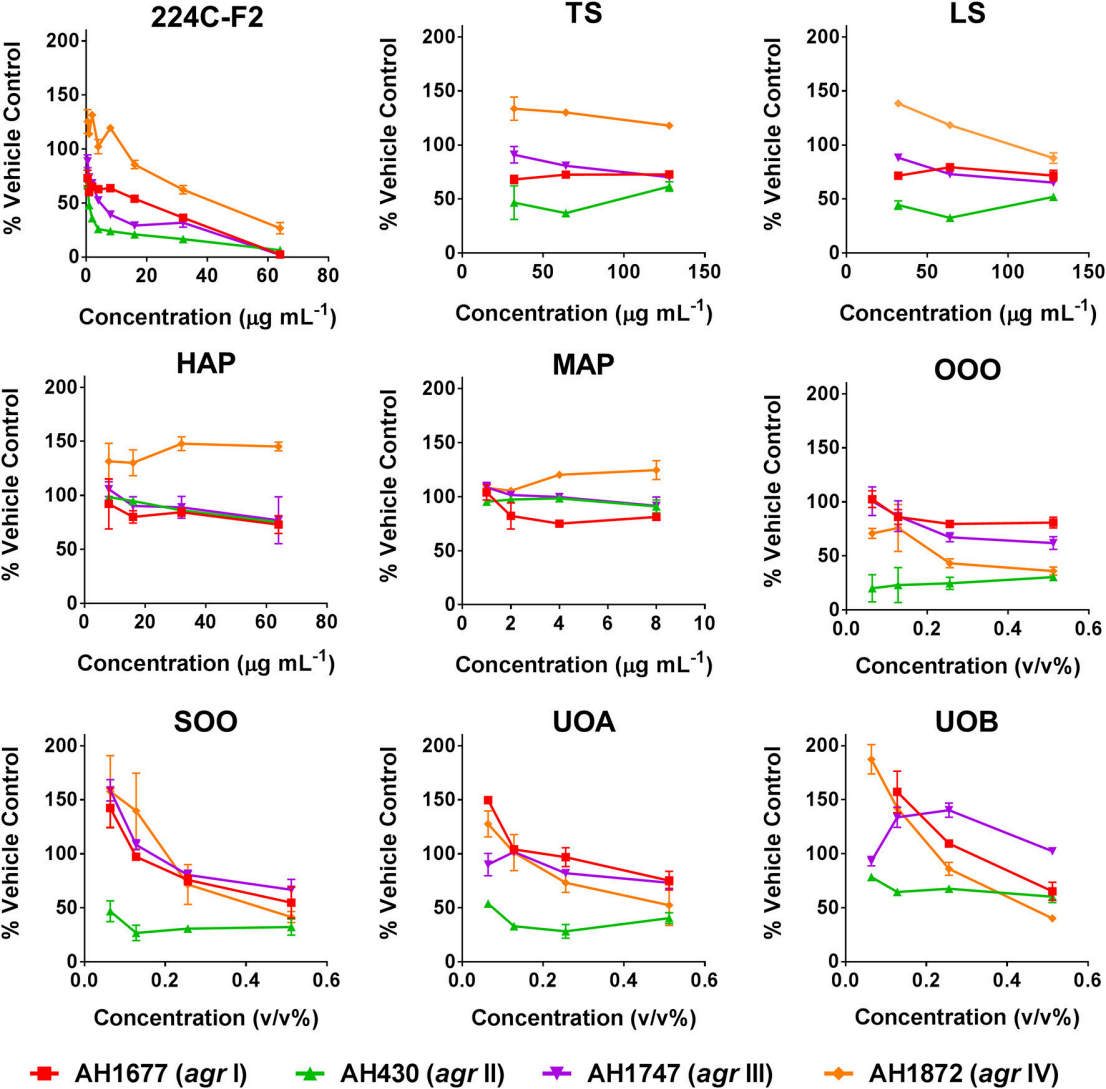
Impact of *H. perforatum* extracts on *S. aureus* quorum sensing, as detected by *agr* reporters. Activity was detected by measure of fluorescence and is reported as percent of the vehicle control. The botanical extract “224C-F2” (a known quorum quencher) was used as the positive control (Quave et al., 2015).

Modest inhibition of quorum sensing (QS) was observed in three (OOO, SOO, UOA) of the oil macerate samples against the *agr*II reporter (AH430), with QSIC_50_ values of 0.064–0.128% v/v, Table 3. All four oil samples inhibited QS in the *agr*IV isolate (QSIC_50_ 0.256–0.512% v/v); SOO also inhibited QS in *agr*I (AH1677) at an IC_50_ of 0.512% v/v. Two of the organic extracts (TS and LS) inhibited quorum sensing (QSIC_50_ of 32 μg mL^−1^ against AH430), but this activity did not exhibit a dose-dependent improvement in activity with an increase in test concentration, Figure 6. Time-dependent examination of OD and relative fluorescence units (RFU) activity over a 20 h period revealed that the observed quorum sensing inhibitory activity was not an artifact of growth inhibitory effects of the samples. Colony counts at 18 h of incubation also confirmed this, with the exception of a minor—but statistically significant—difference in the number of colonies between the olive oil control and SOO and OOO samples for the *agr*II reporter strain and also between the DMSO vehicle control and LS and TS in the *agr*IV reporter strain (Figure 5). None of these exhibited a lower number of colonies in comparison to the vehicle controls in more than one strain.

### Impact on biofilm formation

**Figure 7:**
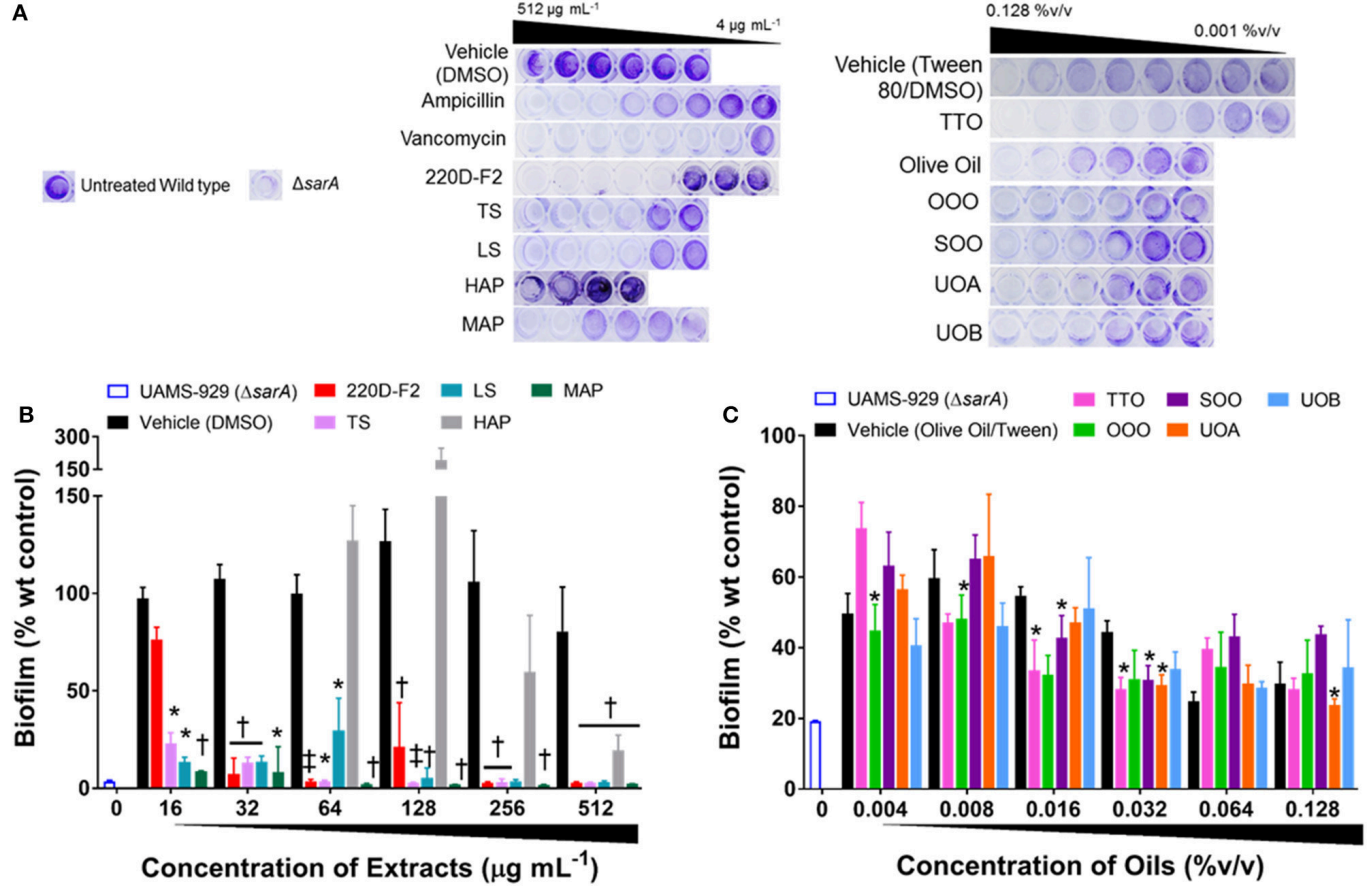
Impact of *H. perforatum* extracts on *S. aureus* biofilm formation, as detected by static microtiter plate crystal violet assay. USA 200 isolate UAMS-1 and its isogenic *sarA* mutant (UAMS-929) were used in the biofilm assay. **(A)** Images of crystal violet stained biofilm in 96-well plates. The optical density (OD_595 *nm*_) of the biofilm eluent is reported as percent of the wild type control (UAMS-1) for the **(B)** organic and **(C)** oil extracts. Significance is denoted as ^*^P < 0.05, *P* < 0.01, and ^‡^*P* < 0.001.

All of the *H. perforatum* extracts (oils and organic extracts) exhibited some degree of inhibition of biofilm formation, Table 3 and Figure 7. Among the organic extracts, MAP was the most potent (MBIC_50_ of 8 μg mL^−1^), however, this extract also exhibited growth inhibitory effects (MIC_50_ of 8 μg mL^−1^). In all of the organic extracts, the growth inhibitory effects of the extracts were responsible for the diminished level of biofilm formed.

With regards to the traditional oil macerates, biofilm inhibition was observed in all four oils (OOO, SOO, UOA, UOB) with MBIC_50_ values ranging from 0.004 to 0.016% v/v. This inhibition was observed in the absence of any growth inhibitory activity (max test concentration of 0.512% v/v).

### Chemical analysis

Percent yields for extracts are reported in Table 1. Analysis of the extracts by HPLC and LC-FTMS revealed the presence of hypericin in the organic and aqueous extracts (MAP, TS, LS, HAP), but not in the oil macerate samples (OOO, SOO, UOA, UOB), Tables 4, 5. Exact mass data of a total of 46 compounds was collected (Table 5).

**Table 4:** Summary of key chemical data.

	**Hyperforin**		**Hypericin**	
	**ESI−**	**ESI+**	**ESI−**	**ESI+**
MAP	+	+	+	−
TS	+	+	+	−
LS	+	+	+	−
HAP	+	+	+	−
OOO	+	−	−	−
SOO	+	−	−	−
UOA	+	+	−	−
UOB	+	−	−	−

*Presence of hyperforin (**14**) or hypericin (**18**) in study samples as determined by mass spectrometry. MAP, methanol extract of flowering aerial parts; TS, commercial tablet supplement; LS, commercial liquid supplement; HAP, aqueous extract of flowering aerial parts; OOO, olive oil macerate of flowering aerial parts; SOO, sunflower oil macerate of flowering aerial parts; UOA, unknown oil A macerate of flowering aerial parts; UOB, unknown oil B macerate of flowering aerial parts; see Table 1 for full details*.

**Table 5:** Mass spectrometry (MS) and MS/MS analysis of the peak data for oil macerates as reported in Figure 8.

**Peak**	**RT (min)**	**RPA**	**m/z**	**Formula**	**Δ ppm**	**MS/MS**	**Putative match**
**OOO**							
12	12.8	0.6	551.37436	C_35_H_51_O_5_	1.3	482.2488, 441.4178, 411.4015, 343.3420, 329.2700, 261.1974	Perforatumone
13	13.4	0.3	**535.3791** [M-H]^−^, 1071.7705 [2M-H]^−^	C_35_H_51_O_4_	0.2	466.3142, 397.3240, 383.3057, 315.2947	Isomer of hyperforin
14	13.8	2.1	**535.3803** [M-H]^−^, 1071.7662 [2M-H]^−^	C_35_H_51_O_4_	2.1	466.3142, 383.3057, 315.2947	Hyperforin
16	14.6	1.0	**277.2169** [M-H]^−^, 555.4411 [2M-H]^−^	C_18_H_29_O_2_	0.7	259.3089, 230.3098	Isomer of linolenic acid
17	15.0	0.2	277.2169, **549.3946**	na	na	480.3090, 397.3433, 329.3007	No matches
21	18.9	46.0	**279.2329** [M-H]^−^, 559.4751 [2M-H]^−^	C_18_H_31_O_2_	1.1	261.3105	Isomer of octadecadienoic acid
24	19.9	3.6	**413.2928**, 633.5133	na	na	395.3275, 369.4152, 343.3766, 275.3397, 261.3305	No matches
34	22.6	0.2	279.2332, 401.2917, 415.3075, **635.5284**	na	na	566.3971, 497.2485, 483.3696, 415.3459, 413.4047	No matches
37	23.0	16.6	**255.2330** [M-H]^−^, 511.4750 [2M-H]^−^	C_16_H_31_O_2_	1.2	237.3247	Isomer of hexadecanoic acid
40	23.7	28.5	**281.2488** [M-H]^−^, 563.5076 [2M-H]^−^	C_18_H_33_O_2_	1.3	281.3895, 263.3492	Isomer of octadecadienoic acid
**SOO**							
12	12.9	0.4	551.3738	C_35_H_51_O_5_	0.9	482.2240, 441.4119, 411.3747, 399.3005, 329.2698, 261.2464	Constitutional isomer of furohyperforin
13	13.5	0.03	535.3788	C_35_H_51_O_4_	0.5	466.3193, 383.2970, 315.2576	Isomer of hyperforin
14	13.9	0.9	535.37933	C_35_H_51_O_4_	1.1	466.3194, 397.3143, 383.2970, 315.2576	Hyperforin
16	14.7	0.6	**277.21695** [M-H]^−^, 555.44167 [2M-H]^−^	C_18_H_29_O_2_	0.7	259.27758, 233.31211	Isomer of linolenic acid
21	19.0	34.9	**279.2329** [M-H]^−^, 559.4750 [2M-H]^−^	C_13_H_31_O_2_	1.1	260.3311, 234.3294	Isomer of octadecadienoic acid
24	20.0	2.9	413.2923, **633.5119**	na	na	589.573	No matches
34	22.7	0.1	415.30800, **635.42920**	na	na	415.4052, 397.3306, 371.3948, 357.4052, 345.4222, 333.1057, 315.3957	No matches
37	23.2	16.3	**255.2330** [M-H]^−^, 511.4749 [2M-H]^−^	C_16_H_31_O_2_	1.2	255.3141, 237.3085	Isomer of hexadecanoic acid
40	23.9	42.3	281.2488 [M-H]^−^, **563.5073** [2M-H]^−^	C_18_H_33_O_2_	1.3	300.1513, 283.1704	Isomer of octadecadienoic acid
**UOA**							
7	9.3	0.27	295.2275, **481.3314**	C_31_H_45_O_4_	0.5	437.4552, 233.1511	Constitutional isomers of hyperibine J
12	12.8	0.18	551.37370	C_35_H_51_O_5_	0.6	482.2713, 412.3792, 411.3806	Constitutional isomers of furohyperforin
13	13.4	0.03	535.37880	C_35_H_51_O_4_	0.5	466.2948, 397.3213, 383.3018, 315.2775	Isomer of hyperforin
14	13.8	1.2	**535.7926** [M-H]^−^	C_35_H_51_O_4_	0.8	466.2948, 383.3018, 315.2775	Hyperforin
16	14.6	0.59 (1.2)	**277.2169** [M-H]^−^, 555.44112 [2M-H]^−^	C_18_H_29_O_2_	0.2	259.3406, 233.3297	Isomer of linolenic acid
21	18.7	34.6	**279.2329** [M-H]^−^, 559.4747 [2M-H]^−^	C_18_H_31_O_2_	0.5	260.3058, 234.3110	Isomer of octadecadienoic acid
24	19.9	5.1	**413.2921**, 633.5118	na	na	343.3614, 275.3439	No matches
29	21.3–23.1	18.0	661.5099, **675.5257**	na	na	657.8013, 631.7033, 420.5482, 303.1914, 255.3595	See peak **19**
36	23.1–23.6	8.9	255.2337, 511.4769, 611.5306, 661.5103, **871.7505**	na	na	853.6913, 829.0806, 255.3568	No matches
40	23.9	25.9	**281.2491** [M-H]^−^, 563.5091 [2M-H]^−^, 897.7645	C_18_H_33_O_2_	1.0	262.3976, 236.2134, 198.1185	Isomer of octadecadienoic acid
**UOB**							
10	11.3	0.31	471.3473	C_30_H_47_O_4_	0.5	423.4483, 405.4416, 393.4567	Astrantiagenin E
12	12.7	0.11	**551.3738**, 675.5210	C_35_H_51_O_5_	0.6	482.2245, 411.3920, 399.3053, 343.3059, 329.2852, 261.2125	Constitutional isomer of furohyperforin
14	13.6	0.17	**535.3789**, 675.5210	C_35_H_51_O_4_	0.8	466.2634, 397.3361, 383.2967, 315.2905	Hyperforin
19	17.3–18.3	2.2	661.5062, **675.5217**	na	na	678.6637, 659.5819, 631.4965, 617.5931	No matches
21	18.7	5.2	**279.2329** [M-H]^−^, 559.4744 [2M-H]^−^, 675.5237, 895.7440	C_18_H_31_O_2_	1.0	260.3058, 234.3120	Isomer of octadecadienoic acid
36	19.2–23.2	22.3	661.5062, **675.5217**	na	na	659.7014, 633.7161, 617.6087	No matches
40	23.5	62.6	**281.2491** [M-H]^−^,563.5091 [2M-H]^−^, 897.7619	C_18_H_33_O_2_	1.2	279.3965, 271.2765, 223.2382	Isomer of octadecadienoic acid
**MAP**							
1	6.0	1.1	569.3866	na	na	500.3059, 431.2675, 383.3261, 347.3187	No matches
3	8.0	7.4	**467.3181** [M-H]^−^, 935.6448 [2M-H]^−^	C_30_H_43_O_4_	0.1	398.4204, 383.4447, 329.3192, 271.2862	Isomer of hyperfirin
4	8.8	1.2	551.3762	C_35_H_51_O_5_	1.6	482.3014, 411.4000, 399.3306, 329.2943	Constitutional isomer of furohyperforin
7	9.2	5.0	**481.3339** [M-H]^−^, 963.6766 [2M-H]^−^	C_31_H_45_O_4_	1.5	412.4372	Constitutional isomer of hyperibine J
12	13.1	2.2	551.3761	C_35_H_51_O_5_	3.6	482.2594, 411.4157	See peak **4**
13	13.5	1.3	**535.3813** [M-H]^−^, 1071.7733 [2M-H]^−^	C_35_H_51_O_4_	2.0	466.3979, 397.4284, 383.4537, 315.4665	Isomer of hyperforin
14	14.0	29.9	**535.3806** [M-H]^−^, 1071.7706 [2M-H]^−^	C_35_H_51_O_4_	2.3	466.4227, 397.4148, 383.4798, 315.4864	Hyperforin
15	14.6	1.0	**549.3972** [M-H]^−^, 1099.8104 [2M-H]^−^	C_36_H_53_O_4_	2.2	480.3700, 411.4376, 397.3677, 329.3505, 313.3432 289.2536	Isomer of adhyperforin
17	15.3	11.2	**549.3970** [M-H]^−^, 1099.8047 [2M-H]^−^	C_36_H_53_O_4_	4.1	480.3942, 411.3993, 397.3756, 329.3478, 313.3410	See peak **15**
18	16.3	1.2	503.0788	C_30_H_15_O_8_	1.0	459.24370	Hypericin
20	17.8	5.9	**535.3812** [M-H]^−^, 1071.7714 [2M-H]^−^	C_35_H_51_O_4_	3.7	397.4702, 275.2561	Constitutional isomer of hyperforin
22	19.3	4.8	**549.3972** [M-H]^−^, 1099.8042 [2M-H]^−^	C_36_H_53_O_4_	4.2	411.4589, 289.2577	See peak **15**
23	19.5	1.1	**535.3809**[M-H]^−^, 1071.7760[2M-H]^−^	C_35_H_51_O_4_	3.2	397.3486, 275.1979	See peak **20**
25	20.6	2.3	543.3368	na	na	515.4505, 473.4408, 432.3971, 405.3768	No matches
26	20.8	2.1	583.3665	na	na	514.3254, 445.4010, 429.4055, 397.3282	No matches
28	21.0	1.1	549.3973, 583.3668, 613.3775, **627.3932**	na	na	609.4827, 558.4290	No matches
30	21.9	1.3	557.3520	na	na	539.4671, 413.4827, 497.4655, 469.4883, 347.3743	No matches
32	22.3	2.6	509.3286	C_32_H_45_O_5_	1.4	481.4579, 465.4379, 439.3066, 371.3111, 327.3694	Isomer of polyprenylated acylphloroglucinol
41	24.1	1.4	597.3812	na	na	539.4692, 469.3719, 455.3666, 399.3432, 343.2971	No matches
43	24.7	2.0	**597.3829**, 611.3987	na	na	455.3906, 399.3456, 343.2941	See peak **41**
**TS**							
2	6.9	2.3	521.0879	C_30_H_43_O_4_	0.1	477.2168	Protopseudohypericin
5	8.9	1.1	519.0724	C_30_H_15_O_9_	0.1	503.3031	Isopseudohypericin
9	11.3	19.7	553.3904	na	na	484.3227, 415.3904, 401.3363, 338.2878	No matches
12	13.1	6.1	551.3752	C_35_H_51_O_5_	1.5	482.3489, 455.4689, 399.3075, 330.2504	Constitutional isomer of furohyperforin
13	13.4	2.4	535.3799	C_35_H_51_O_4_	0.6	466.2862, 397.3600, 383.3395, 315.3039, 275.2309	Isomer of hyperforin
14	14.1	5.0	**535.3810** [M-H]^−^, 1071.7718 [2M-H]^−^	C_35_H_51_O4	3.2	466.2948, 383.3485, 315.3024	Hyperforin
17	15.2	6.1	**549.3968** [M-H]^−^, 1099.8028 [2M-H]^−^	C_36_H_53_O_4_	1.9	480.3235, 397.3463, 329.3124	Isomer of adhyperforin
18	16.4	2.4	**503.0778**, 551.3753	C_30_H_15_O_8_	0.5	503.2111, 459.2324	Hypericin
20	17.7	5.0	**535.3807** [M-H]^−^, 1071.7719 [2M-H]^−^	C_35_H_51_O4	2.8	397.3674, 275.2193	Constitutional isomer of hyperforin
22	19.1	2.3	549.3966	C_36_H_53_O_4_	3.0	411.3998, 289.2541	See peak **17**
23	19.4	1.9	**535.3806**, 559.4747	C_35_H_51_O_4_	3.0	397.3598, 275.2287	See peak **20**
26	20.8	3.5	583.3658	na	na	539.3626, 495.3746, 471.4266, 455.3294, 441.3578, 427.3751, 385.3207, 345.3529, 329.3006, 275.2133	No matches
28	21.0	1.5	627.3940	na	na	595.5207, 567.5077, 558.3551, 484.4406	No matches
32	22.3	1.9	509.3286	C_32_H_45_O_5_	1.7	481.4253, 465.3411, 439.3995, 398.3324, 371.3304	Isomer of polyprenylated acylphloroglucinol
33	22.5	0.4	643.3877	na	na	625.3742, 599.4978, 583.5160, 574.4302, 505.3105	No matches
37	23.6	33.0	511.4748	na	na	435.4601	No matches
43	24.7	3.7	611.3991	na	na	593.5766, 551.4913, 542.3932	No matches
45	31.8	1.9	**283.2642** [M-H]^−^, 567.5364 [2M-H]^−^	C_18_H_35_O_2_	0.1	265.3776	Isomer of octadecanoic acid
**LS**							
1	5.7	1.6	569.3851	na	na	273.1406, 257.1530, 229.1662, 179.0143, 151.0151	No matches
4	8.8	0.7	551.3753	C_35_H_51_O_5_	1.0	482.2749, 411.3920, 383.3228, 329.2915, 275.2239	Constitutional isomers of furohyperforin
8	10.3	1.1	569.3854	na	na	na	No matches
9	11.1	1.2	553.3907	na	na	485.3576, 416.3804, 402.3450, 334.2963	No matches
11	11.8	1.6	467.3170	C_30_H_43_O_4_	0.3	398.2772, 329.2860, 287.2419, 275.2608, 219.1733	Isomer of hyperfirin
12	12.9	2.4	551.3753	C_35_H_51_O_5_	2.2	482.2471, 411.3996	Constitutional isomer of furohyperforin
13	13.2	1.3	**535.3805** [M-H]^−^,1071.7709 [2M-H]^−^	C_35_H_51_O_4_	1.2	466.3801, 397.3877, 383.3733, 315.3414, 275.2349	Isomer of hyperforin
14	13.8	28.2	**535.3810** [M-H]^−^, 1071.7718 [2M-H]^−^	C_35_H_51_O_4_	3.3	466.4120, 383.3845, 315.3482	Hyperforin
15	14.4	1.2	**549.3965** [M-H]^−^, 1099.8062 [2M-H]^−^	C_36_H_53_O_4_	1.6	480.2871, 411.3738, 397.3655, 329.3298, 313.3076, 289.2431	Isomer of adhyperforin
17	15.0	11.4	**549.3970** [M-H]^−^, 1099.8034 [2M-H]^−^	C_36_H_53_O_4_	3.7	480.2986, 397.3735, 329.3245	See peak **15**
18	16.2	1.3	503.0778	C_30_H_15_O_8_	0.6	487.2185, 459.2460	Hypericin
20	17.6	8.7	**535.3809** [M-H]^−^, 1071.7711 [2M-H]^−^	C_35_H_51_O_4_	3.0	397.4060, 275.2363	Constitutional isomer of hyperforin
22	19.0	4.7	**549.3966** [M-H]^−^, 1099.8046 [2M-H]^−^	C_36_H_53_O_4_	3.1	411.3572, 289.2496	see peak **15**
23	19.2	3.3	**535.3808** [M-H]^−^, 1071.7730 [2M-H]^−^	C_35_H_51_O_4_	2.9	397.3746, 275.2188	See peak **20**
27	20.7	1.8	**549.3967** [M-H]^−^, 1099.8068 [2M-H]^−^	C_36_H_53_O_4_	3.5	411.3706, 289.2387	See peak **15**
31	22.1	1.8	509.3289, **583.3661** [M-H]^−^, 637.4499, 1167.7499 [2M-H]^−^	na	na	539.4567, 471.4214	No matches
35	23.0	3.6	**367.2647** [M-H]^−^, 735.5381 [2M-H]^−^	na	na	177.1379	No matches
38	23.3	0.9	523.3455, **597.3814**, 815.5020	na	na	553.4625, 528.3395, 495.4520, 471.4037	No matches
39	23.9	1.3	**255.2331** [M-H]^−^, 511.4743 [2M-H]^−^	C_16_H_31_O_2_	0.2	242.1994	Hexadecanoic acid
43	24.7	2.8	551.3789, 597.3834, **611.4005**, 833.6381, 1163.8008	na	na	na	No matches
44	25.6	1.1	**535.3809**, 625.4123	C_35_H_51_O_4_	2.9	397.3663, 275.2371	See peak **20**
46	33.2	1.2	**551.3754** [M-H]^−^, 1103.7625 [2M-H]^−^	C_35_H_51_O5	2.2	508.5036, 456.4861	See peak **12**
**HAP**							
3	7.7	7.0	**467.3162**, 567.3691, 935.6430	C_30_H_43_O_4_	0.4	398.2960	Isomer of hyperfirin
6	8.9	7.3	**481.3326** [M-H]^−^, 963.6747[2M-H]^−^	C_31_H_45_O_4_	0.3	412.3196	Constitutional isomer of hyperibine J
12	12.8	3.2	551.3746	C_35_H_51_O_5_	0.4	482.2764, 413.3273, 383.3141, 329.2978	Constitutional isomer of furohyperforin
14	13.8	35.7	**535.3810** [M-H]^−^, 1071.7711 [2M-H]^−^	C_35_H_51_O_4_	1.7	466.2689, 451.3754, 383.3375, 315.3061	Hyperforin
15	14.3	0.6	549.3955	C_36_H_53_O_4_	0.6	480.2824, 397.3177, 329.3075, 313.2955, 289.2541	Isomers of adhyperforin
17	15.0	15.6	**549.3967** [M-H]^−^, 1099.8029 [2M-H]^−^	C_36_H_53_O_4_	3.3	480.2938, 397.3234, 329.3068	Isomer of adhyperforin
18	16.2	1.5	503.0774	C_30_H_15_O_8_	0.2	487.1872, 459.2382	Hypericin
23	19.2	0.8	535.3800	C_35_H_51_O_4_	1.3	397.34501, 275.19977	Constitutional isomer of hyperforin
25	20.3	2.1	529.3198, **543.3355**	na	na	515.4520, 499.4560, 473.4428, 432.3554, 405.3853	No matches
28	20.8	1.0	627.3928	na	na	609.5030, 581.5090, 567.5005, 558.3908	No matches
30	21.6	1.7	543.3355, **557.3512**	na	na	na	No matches
32	22.1	3.3	509.3287	C_32_H_45_O_5_	1.4	481.4410, 465.4290, 439.3782, 398.3118, 371.3374	Isomer of polyprenylated acylphloroglucinol
38	23.3	1.3	523.3443	C_33_H_47_O_5_	1.4	495.4596, 479.4419, 439.4268, 412.3792, 385.3166, 369.4376, 341.3946, 329.3829	Isomer of polyprenylated bicyclo[3.3.1]nonene
42	24.4	1.2	613.3777, **627.3943**	na	na	583.4786	No matches
43	24.6	4.1	597.3826, **611.4005**, 807.6203	na	na	552.5200, 551.4894	No matches

*RT, retention time; RPA, relative percent abundance, based on peak area. Bold m/z values are parent ions for the reported MS/MS fragments when more than one ion present*.

**Figure 8:**
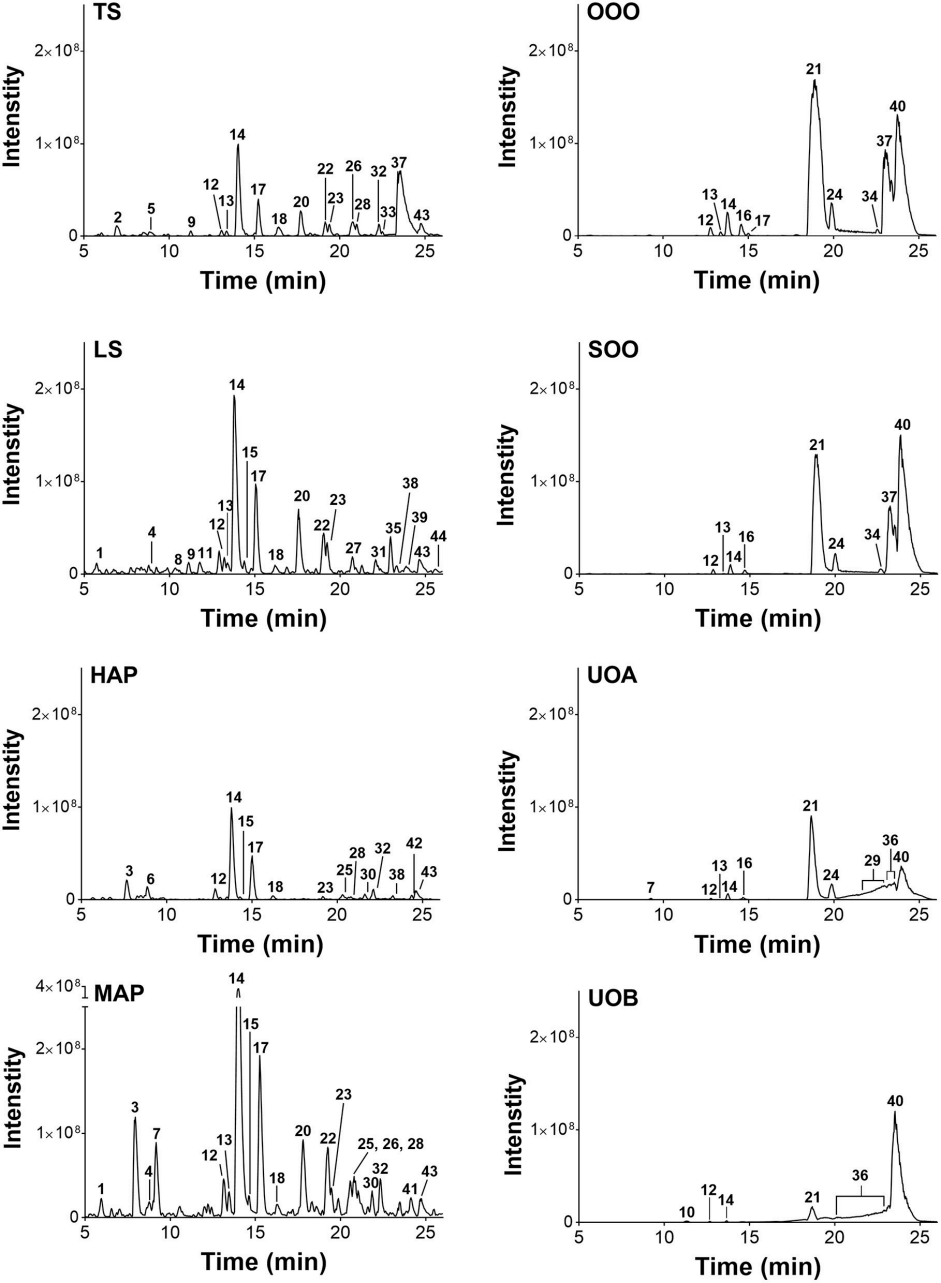
Characterization of *H. perforatum* extracts by LC-FTMS. Chromatograms of organic and oil extractions of *H. perforatum* are reported; peak numbers correspond to Table 5.

LC-FTMS analysis of the oil samples revealed the presence of 12 distinct compounds, only four of which were found in all four samples, Figure 8; this included the putative compounds: constitutional isomer of furohyperforin (**12**), hyperforin (**14**), and isomers of octadecadienoic acid (**21** and **40**), Table 5. Compounds **21** and **40** had the highest relative abundance of all components of the oil macerate samples. Peaks **13, 14, 20, 23**, and **44** all had m/z of 535.38, by comparison of the retention times and mass spectra with that of an authentic standard it was determined that **14** was hyperforin. Peaks **13** and **14** also have similar MS^2^ fragments. Since hyperforin has been identified as having 3 tautomers, 1,3 diketone and two enols, the authors have assigned **13** as the enol form. This tautomerization was shown to occur on HPLC columns at a pH of 2.5, very similar to the pH of the 0.1% formic acid mobile phases used in these HPLC methods (Fourneron and Naït-Si, 2006; Lee et al., 2006). Peaks **20**, **23**, and **44** all have base peaks with m/z 535.38; however, the MS^2^ is not consistent with hyperforin and these peaks have been assigned as constitutional isomers of hyperforin.

Analysis of the organic and aqueous samples revealed the presence of six compounds common in all four samples: **12, 14**, isomer of adhyperforin (**17**), hypericin (**18**), hyperforin (**23**), and **43**; compounds **12** and **14** were the only ones also present in the oil samples. Peak **20** (an isomer of hyperforin) was present in all of the organic extracts (TS, LS, MAP), but absent in the aqueous extract (HAP).

## Discussion

### Chemistry

Through LC-FTMS analysis of different *H. perforatum* formulations, we have demonstrated that the traditional oil macerate (Oleum Hyperici), used in topical applications for skin and soft tissue infections throughout the Balkans, contains the bioactive compound hyperforin, but not the photosensitizing compound, hypericin. In contrast, the MeOH, aqueous, and commercial supplement extracts all featured both hypericin and hyperforin. The composition difference between the two classes of sample in this study is most likely due to the methods of their extractions, with photo-extraction in an oil solution yielding very different compounds than a low-light organic or aqueous extract. This supports field observations of a lack of reported photosensitive reactions among users of the oil for topical skin care.

However, in contrast to our findings concerning the chemistry of Oleum Hyperici, Miraldi et al. (2006) presented a different case, in that hyperforin, adhyperforin, hyperevolutin A and B, hypericin and pseudohypericin are entirely absent from preparations of this oil macerate. These results were found in oil macerates produced by 15 days of sun exposure—as opposed to 40, as described in ethnobotanical research (Mustafa et al., 2015). Nevertheless, the presence of hyperforin—a photolabile, oxidation prone phloroglucinol derivative—in a formulation which relies on extended periods of sun exposure presents some additional questions. Mainly, how is it possible that this compound remains stable in this traditional preparation? One potential explanation based on the work of Boskou (1978) might be that the abundant terpenoids in the oil itself could be stabilized in light and heat, and in turn, act as sacrificial reducing agents for the ROS preventing the eventual oxidation of hyperforin into furohyperforin. While this change can be evaluated by an increase in the oil's viscosity due to an increase ratio of palmitic to linoleic acid, further experimentation and research is required to evaluate this change in the oil. The degradation of hyperforin follows first order kentics yielding a tautomeric mixture of 2-methyl-3-hydroxy-4-(1-oxo-2-methyl-1-propyl)-1,5-dioxo-6-(3methyl-1-but-2-enyl)-2-cyclohexeneand 2-methyl-1,3,5-trioxo-4-(1-hydroxy-2-methyl-1-propylene)-6-(3-methyl- 1-but-2-enyl)-cyclohexane via three Norrish type II reactions (D'Auria et al., 2008). The oil or other compounds in the oil macerate may be absorbing some of the light necessary for the Norrish reactions to occur, thus preventing the hyperforin from decomposing. An analysis of a purchased olive oil sample by UV-Vis showed a strong absorption band at 299–302 nm (data not shown) and the spectrum below this wavelength was very noisy indicating that the oil itself has a strong and complex absorbance in this range. Published UV-Vis spectra of hyperforin show a λ of 298–310 nm depending on the solvent (Vuong et al., 2011; Ng et al., 2017). Based on this analysis, the olive oil itself is absorbing UV light in the range that is necessary to degrade the hyperforin and thus protecting the compound in the oil maceration.

Other factors that can contribute the variations in the oil macerates include the natural variation in the *H. perforatum* plant material used to make the preparations in the different studies. These variations are known to influence both the chemical composition and bioactivity of *Hypericum* products (Marrelli et al., 2014). Variations in secondary metabolite concentrations could be the result of the botanical source material being grown in differing climates or having been exposed to differing stressors, such as herbivory, infection, or drought. Due to such potential variation, any commercial preparation of *H. perforatum* should be standardized to established marker compounds using accepted analytical methods, those published by AOAC [AOAC Stakeholder Panel on Strategic Foods Analytical Methods (SPSFAM), 2013].

### Bioactivity

We demonstrated with our bioactivity findings that while the traditional oil macerates (Oleum Hyperici) do not exhibit strong anti-staphylococcal growth inhibitory activity as is noted in the organic and aqueous preparations of the flowering aerial parts, they do exhibit biofilm inhibitory properties at sub-inhibitory concentrations for growth and exhibit modest quorum quenching effects against three of the four accessory gene regulator (*agr*) alleles. The quorum sensing inhibitory activity of the ethyl acetate extract of a related species (*H. connatum*) has been reported against *Pseudomonas aeruginosa* (Fratianni et al., 2013). In previous work, we assessed the capacity of *H. perforatum* and many other Mediterranean species to inhibit production of delta-hemolysin, a transcriptional product controlled by the *agr* system, however the ethanolic extract of *H. perforatum* stems exhibited only mild inhibitory activity (36% inhibition of delta-toxin production; Quave et al., 2011). Thus, to the best of our knowledge, this is the first report of a *Hypericum* species extract or formulation inhibiting quorum sensing at a level >50% against *S. aureus*.

The growth inhibitory activity of the extracts containing hypericin (MAP, TS, LS, HAP) was unsurprising as hypericin is reported to be the major growth inhibitory antibacterial agent for this species (Saddiqe et al., 2010; Yow et al., 2012). In addition to numerous studies on the growth inhibitory properties of *H. perforatum* extracts, a few have also examined their anti-biofilm potential. Extracts of the adventitious roots have demonstrated anti-biofilm activity against the fungus *Malassezia furfur* (Simonetti et al., 2016). With regards to anti-biofilm activity in bacteria, hyperforin and its hydrogenated analog have been shown to inhibit planktonic and biofilm cultures of *S. aureus* and *E. faecalis* (Schiavone et al., 2013). A suite of *Hypericum* spp. secondary metabolites were found to exhibit anti-biofilm activities against *S. aureus* and *S. epidermidis*, one of which—a phloroglucinol from *H. punctatum*—had an MBIC (no detectable biofilm formation) of just 1.95 μg mL^−1^ (Sarkisian et al., 2012).

In our analyses, we found that the organic, aqueous, and oil extracts all exhibited some level of biofilm inhibition against *S. aureus*. However, it was difficult to gain an accurate assessment of the inhibitory activity for biofilm formation in the organic and aqueous extracts (HAP and MAP) as this was confounded by their strong growth inhibitory activity. On the other hand, the oil macerates (OOO, SOO, UOA, UOB), which lacked the strongly antibacterial hypericin, did demonstrate statistically significant inhibition of biofilm formation in the absence of growth inhibition. Early work with *H. perforatum* attributed antibacterial activity to hyperforin which was present in all the oil macerates (Gurevich et al., 1971). While hypericin has stronger antibacterial activity, hyperforin has reported MICs and minimum bactericidal concentrations (MBC) as low as 1 mg mL^−1^ by agar-diffusion assays for some preparations against *S. aureus* (Schempp et al., 1999). Based on studies concerning the anti-biofilm effects of hyperforin (Schiavone et al., 2013), its MIC, and our documentation of appreciable levels of hyperforin in the traditional oil macerate, could explain—at least in part—the activity observed. Importantly, while other studies have described the anti-biofilm activity of isolated compounds and other *Hypericum* extracts, this is the first report of the biofilm inhibitory activity of the traditional Oleum Hyperici formulation. This data concerning the antibacterial (anti-biofilm and quorum quenching) activity combined with the absence of the harmful photosensitizing agent hypericin, provide compelling evidence of valid efficacious and safe use of this traditional remedy.

## Conclusions

Different formulations of *H. perforatum* flowering aerial parts are used in traditional medicine, Western herbalism, and in dietary supplements. In this study, we aimed to investigate the safety and antibacterial efficacy of an oil macerate formulation (Oleum Hyperici), which is one of the most common topical therapies for skin and soft tissue infections used in the Balkans as compared to other organic and aqueous extractions, and commercial supplement preparations of the plant. We determined that the traditional preparation of Oleum Hyperici, which involves 40 days of sun exposure in oil, results in a product that lacks the phototoxic naphthodianthrone compound hypericin, responsible for skin sensitization reactions and hypericism, supporting our hypothesis. Our hypothesis concerning the antibacterial activity of Oleum Hyperici was refuted in some assays, and supported in others. Specifically, oil macerates did not inhibit bacterial growth overall, but did significantly inhibit biofilm formation and quorum sensing, which is responsible for the recalcitrant nature of *S. aureus* infections and the regulation of a suite of harmful staphylococcal toxins, respectively. This suggests that the traditional Oleum Hyperici formulation may have more utility in regulation of staphylococcal virulence and pathogenesis rather than classic antibiotic activity.

In conclusion, we have demonstrated that this topical folk-medical therapy for skin and soft tissue infections (including ulcers and wounds) could represent a safe and efficacious therapy for further development. Perhaps the most important consequence of this study is the chemical and biological validation of a traditional medicine, which could continue to play an important role in human medicine in the future. From a union of ethnobotany and biochemical analyses, these findings corroborate the biological mechanism of efficacy of this treatment.

## Author contributions

AH, BM, and CQ: Collected the material; JL and CQ: Conceived and designed the experiments; AB, AK, CQ, JL, and KN: Performed the experiments and analyzed the data; AK, JL, and CQ: Prepared the draft; All authors proofread the final draft and approved the final manuscript.

### Conflict of interest statement

The authors declare that the research was conducted in the absence of any commercial or financial relationships that could be construed as a potential conflict of interest.
